# Characterization of the complete mitochondrial genome of yellow stripe mutant in onion (*Allium cepa* L.)

**DOI:** 10.1080/23802359.2020.1797547

**Published:** 2020-07-27

**Authors:** Linchong Hui, Wei Chen, Meihong Pan, Weiya Li, Linyu He, Jiangying Wang, Meihua Miao, Haifeng Yang

**Affiliations:** Lianyungang Academy of Agricultural Sciences, Lianyungang, People’s Republic of China

**Keywords:** *Allium cepa* L., mitochondrial, yellow stripe mutant

## Abstract

The complete onion of yellow stripe mutant tissues mitochondrial genome was determined and analyzed in this study. The complete sequence length is 324,182 bp, including 37 CDS, 13 tRNA, and G + C content percentage of 45. Compared with the normal onions, 556 SNPs, 279 small indel numbers were detected, and the mutations of 14 SNPs and two small indels in the exon region affected the translation of proteins. Phylogenetic tree analysis showed that yellow stripe mutant onion is closely clustered with other *Allium cepa* L. The mutant of onion plays an important role in developing molecular marker-assisted breeding.

The onion is a great genus of monocotyledonous plants in the family Amaryllidaceae (Chase et al. 2009). Yellow stripe mutant of Allium (onion, garlic) leaf belongs to maternal inheritance (Tatebe 1961, 1968). The higher the degree of yellow in the mutant tissue in phenotypic characteristics, the weaker the plant. The yellow stripes are accompanied with tubular leaves, calyxes, flower buds, peduncles, petals, pistils, and stamens in the umbel throughout the growth period, until they wither and do not turn green (Yang et al. 2019). Maintainer strains can be used as morphological markers for onion cytoplasmic male cross breeding and identification of transgenic plants.

The material of this study is the yellow tissue of the onion in China. Fresh leaves of onion were collected from Lianyungang City, Jiangsu Province (N34°32′42″, E119°12′10″). Voucher specimen was deposited in the Lianyungang Academy of Agricultural Sciences (under collection numbers of LAAS20170003). DNA was provided to construct an Illumina PE library (400 bp). After quality control of the obtained sequencing data, bioinformatics analysis was utilized to complete the sample's mitochondrial genome de novo splicing and sequencing analysis. The published onion mitochondrial genome was used as reference (KU318712.1) (Kim et al. 2016), based on GATK correction comparison results. GATK software was used to detect SNP, small indel again, and the sequencing depth, comparison quality was filtered out. The complete sequence length is 324,182 bp, including 37 CDS, 13 tRNA, and G + C content percentage of 45. The lower loci were 556 SNPs and 279 small indel numbers with high confidence. SNPs were obtained using Annovar program combined with annotation information of onion mitochondrial genome: downstream (4), exonic (14), intergenic (41), intronic (478), ncRNA_exonic (8), and upstream (11). Small indel: downstream (3), exonic (2), intergenic (18), intronic (250), ncRNA_exonic (3), and upstream (3). For 14 SNPs and two small indel sites in the exonic region, the mutation sites will have an effect on protein translation. Structural variants (SVs) detection types include: two deletions (DEL), seven insertions (INS), seven inversions (INV), four ITXs (intra-chromosomal translocation (ITX)), a total of 20 SVs point. Relevant data are uploaded in NCBI (GenBank accession number MT240856.1).

Currently, very few genomes were available for Liliopsida and not for Asparagales or Amaryllidaceae, except for only two onion mitochondrial genomes (KU318712.1 and AP018390.1). We use the published mitochondria of Liliaceae plants in the database as the mitochondrial genes of different plants are quite different, some species may lack a certain protein sequence and the existence of multiple copies, resulting in a small number of single-copy homologous protein sequences that are finally identified. Hence, ignore the single copy and use orthofinder (v2.3.5) software to obtain homologous proteins, align these protein sequences (MAFFT v7.427), and use trimal (v1.4.1) to trim the aligned sequences, concatenate these sequences finally, use RAxML 8.2.10 to build the evolution tree, select PROTGAMMAJTT as the model with bootstrap = 1000 (Figure 1). The NJ tree shows that yellow stripe mutant onion is closely clustered with other *Allium cepa* L. Complete mitochondrial genome data provide basic information for onion molecular marker development.

**Figure 1.**
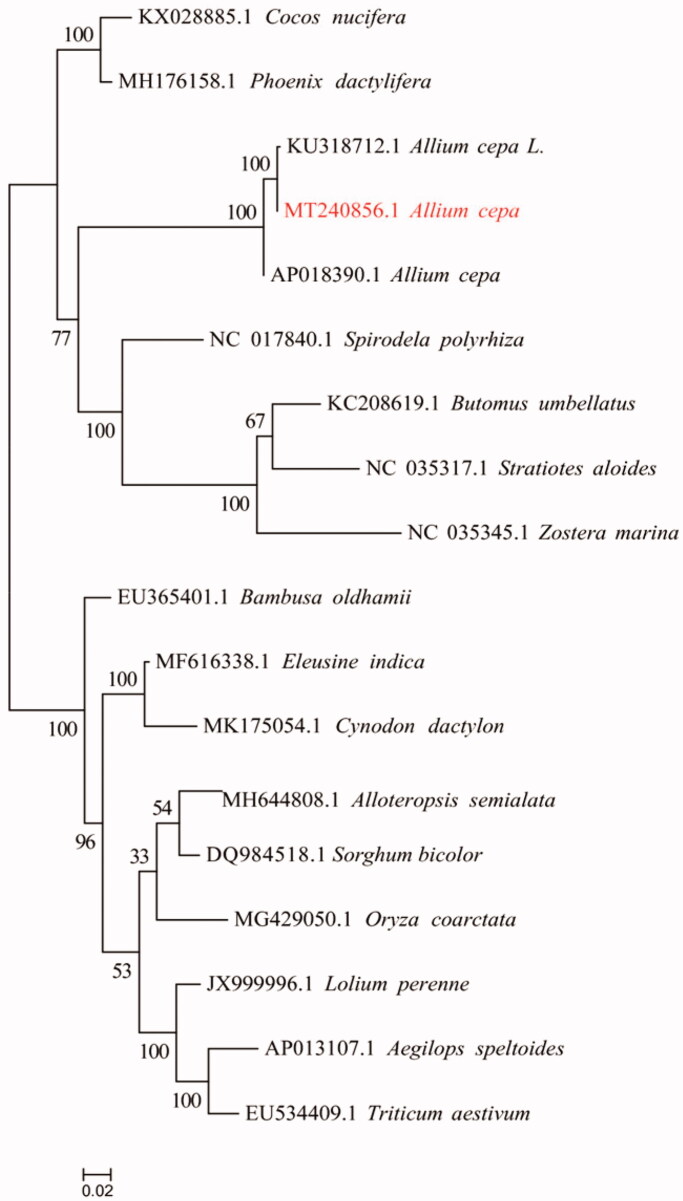
Phylogenetic tree reconstruction of 18 taxa based on the mitochondrial homologous proteins.

## Data Availability

The data that support the findings of this study are openly available in National Center for Biotechnology Information at https://www.ncbi.nlm.nih.gov/, accession number MT240856.1.
